# A Traditional Herbal Formula Xianlinggubao for Pain Control and Function Improvement in Patients with Knee and Hand Osteoarthritis: A Multicenter, Randomized, Open-Label, Controlled Trial

**DOI:** 10.1155/2018/1827528

**Published:** 2018-02-12

**Authors:** Fei Wang, Lei Shi, Yaonan Zhang, Kunzheng Wang, Fuxing Pei, Hanmin Zhu, Zhanjun Shi, Tianzun Tao, Zhihua Li, Ping Zeng, Xiaobing Wang, Quan Ji, Ling Qin, Qingxun Xue

**Affiliations:** ^1^Department of Orthopedics, Beijing Hospital, National Center of Gerontology, Beijing 100730, China; ^2^Department of Orthopedics, The Second Affiliated Hospital, Medical School of Xi'an Jiaotong University, Xi'an 710004, China; ^3^Department of Orthopaedics, First University Hospital, West China University of Medical Sciences, Chengdu 610041, China; ^4^Department of Osteoporosis, The Affiliated Huadong Hospital of Fudan University, Research Unit of Elderly Bone Metabolic Disease of Shanghai Geriatrics Institute, Shanghai 200040, China; ^5^Department of Orthopaedics, Southern Medical University, Nanfang Hospital, Guangzhou 510515, China; ^6^Second Department of Orthopaedic Surgery, The Affiliated Second Hospital of Harbin Medical University, Harbin 150086, China; ^7^Institute of Integrative Medicine, Hebei Medical University, Shijiazhuang 050017, China; ^8^Beijing Institute of Geriatric Diseases, Beijing Hospital, National Center of Gerontology, Beijing 100730, China; ^9^Department of Orthopaedics & Traumatology, The Chinese University of Hong Kong, Shatin 200433, Hong Kong

## Abstract

Evidence of efficacy of a traditional herbal formula Xianlinggubao (XLGB) for treatment of osteoarthritis (OA) is limited. The present study was designed to evaluate the efficacy of XLGB in the management of patients with knee and hand OA. This was a multicenter, stratified, open-label, randomized controlled trial conducted at six centers in China. People aged 40 or above, diagnosed with OA of the knee or hand, were randomly assigned to the XLGB treatment group or watchful waiting control group. Main outcome measures were the changes in the numeric pain rating scales (NPRS) and the Western Ontario and McMaster Universities Arthritis Index (WOMAC) or the Australian/Canadian Osteoarthritis Hand Index (AUSCAN) scores, from baseline to 6 months. In total 534 patients (272 to XLGB and 262 to control group) received interventions. Participants in the XLGB group exhibited significant improvement in NPRS (*P* < 0.001) and WOMAC score (*P* < 0.001) or AUSCAN score (*P* < 0.001) compared to control group. Treatment with XLGB at current regime significantly reduced pain and improved function of the knee and hand in patients with OA over a 6-month period, implying that XLGB could be suggested as an alternative treatment for patients with knee or hand OA.

## 1. Introduction

Osteoarthritis (OA) is one of the most common forms of chronic arthritis, causing pain, functional limitation, and disability. Worldwide estimates are that 9.6% of men and 18.0% of women over the age of 60 years have symptomatic osteoarthritis [1]. As the population is aging, the incidence of OA will continue to rise. OA is characterized by degeneration of the joints and usually affects the knees, hips, and hands. The symptoms of OA most commonly involve pain in the affected joint(s), associated with swelling, stiffness, and creaking of the joint [2, 3].

No effective cure or disease-modifying treatments are currently available for OA [4–6]. Current pharmacological treatments focus on reduction of pain and increased mobility to improve overall quality of life, including nonsteroidal anti-inflammatory drugs (NSAIDs), opioid analgesics, and intra-articular steroid injection [4–6]. However, the use of these drugs may prove ineffective in some patients [7] and is frequently associated with adverse effects [8, 9]. Hence it is desirable to develop new or test alternative drugs with good efficacy and less or no adverse effects in the treatment of OA, specifically for those patients who do not respond well to conventional medical therapy. Accordingly, many patients therefore also turn to complementary and alternative medicines, such as herbal supplements.

In oriental counties, herbal medicines have been commonly used to treat OA for centuries [10, 11]. Xianlinggubao (XLGB) is a type of traditional Chinese medicines (TCM) that is widely used for treatment of bone disorders [12–15]. The XLGB capsule was officially approved by the China Food and Drug Administration (CFDA) in 2002 as the over-the-counter (OTC) drug for treatment of osteoarthritis, osteoporosis, aseptic osteonecrosis, and bone fracture (CFDA, China, Z20025337). It has been used for clinical applications over 15 years and clinical trials demonstrated its efficacy and safety for prevention of postmenopausal osteoporosis [13, 14, 16]. As emerging evidences suggest that the course of primary OA is associated with osteoporosis, especially at subchondral bone [17, 18], herbal Fufang might also have preventive effects on OA prevention via inhibition of bone loss.

However, despite a wide prescription in our OA clinics, evidence for efficacy and safety of XLGB for OA is limited [19]. Therefore, robust randomized controlled trial (RCT) is desirable and supported with China governmental funding body to conduct this multicenter, randomized controlled trial to evaluate the efficacy and safety of XLGB in the management of knee and hand OA.

## 2. Methods

### 2.1. Trial Design

This was a multicenter, stratified, open-label, parallel-group controlled trial with two arms conducted in China, to exam the efficacy and safety of XLGB herbal Fufang for management of patients aged 40 years or above with clinically diagnosed OA at knee and/or hand.

The study was conducted according to the principals of good clinical practice and the principles of the Declaration of Helsinki. The protocol was approved by the Regional Committee for Medical and Health Research Ethics at each participating center (reference number 2005/001). All eligible participants signed informed consent before entering the randomization for this perspective clinical trial.

### 2.2. Settings and Participants

The study was conducted at six CFDA certified medical centers in China, including Shi Jiazhuang, Harbin, Shanghai, Guangzhou, Chengdu, and Xi'an. In every center, participants were all Han nationality aged 40 years or above who were screened for eligibility via clinical symptoms, physical examinations, and radiological examinations. An interviewer-controlled questionnaire was conducted to collect information on sociodemographic features, lifestyle information such as occupation, habit of smoking, alcohol consumption, family history, medical history, physical activity, and joint pain and function. Radiological examinations included bilateral knees at anteroposterior and lateral views and bilateral hands at an anteroposterior view.

The inclusion criteria were adults aged 40 or above, diagnosed with primary OA of the knee or hand according to the American College of Rheumatology clinical criteria [20, 21], combined with definite radiological changes of OA (Kellgren and Lawrence grade [K-L grade] [22] ≥2) at the index joint, the numeric pain rating scales (NPRS) [23] equal or more than 2, and the Western Ontario and McMaster Universities Arthritis Index (WOMAC) [24] ≥20 or the Australian/Canadian Osteoarthritis Hand Index (AUSCAN) [25] ≥15.

Exclusion criteria were as follows: having neurological condition that could significantly affect the index joint, history of upper gastrointestinal ulcers within 6 months before screening, history of gastrointestinal hemorrhage within 12 months prior to the screening, renal or hepatic impairment, diseases of the blood system, uncontrolled hypertension, severe heart disease, or DM with poor blood glucose control. Female participants in pregnancy, lactation, or planned pregnancy during the study were also excluded.

### 2.3. Randomization and Interventions

Eligible patients were randomized at baseline according to a computer-generated randomization schedule, which was prepared by the study biostatistician. Randomization was stratified by study center, sex, and age (<60 versus ≥60) to ensure balance between intervention groups. Patients and the study team were blinded to treatment allocation until all patients had completed all baseline measurements, and interventions were assigned. This study was designed as open-label because of financial considerations.

Eligible patients were randomly assigned to the experimental or watchful waiting control group at a 1 : 1 ratio. The subjects in the experimental group were given 3 g of XLGB capsule (1.5 g, twice daily) for a total of 6 months. Compliance with prescribed treatment was assessed by counting the numbers of unused capsules at last visit (month 6). The subjects in the control group were watched without receiving specific treatment but were allowed to take orally rescue painkillers, and the patients who were still intolerant of pain were allowed to withdraw from the trial and receive more aggressive treatment. During the entire period of the clinical trial, patients with a severe level of pain were allowed to take the rescue medicine (analgesic), and patients who had adverse effects were reported and documented. A patient diary was applied to record dosing details and rescue medicine used. Patients were withdrawn due to adverse treatment effects. The study end point was set at 6 months.

XLGB capsules (0.5 g/capsule) were provided by the Tongjitang Chinese Medicines Company. As herbal Fufang, XLGB capsule consists of six herbs with percentages in weight as follows: Herba Epimedii (*Epimedium brevicornum* Maxim, Yinyanghuo) (70%), Radix Dipsaci (root of* Dipsacus asper* Wall. ex C. B. Clarke, Xuduan) (10%), Radix Salviae Miltiorrhizae (root and rhizome of* Salvia miltiorrhiza* Bunge, Danshen) (5%), Rhizoma Anemarrhenae (rhizome of* Anemarrhena asphodeloides* Bunge, Zhimu) (5%), Fructus Psoraleae (fruit of* Psoralea corylifolia* L., Buguzhi) (5%), and Radix Rehmanniae (root of* Rehmannia glutinosa* (Gaertn.) DC, Dihuang) (5%) [14, 26].

### 2.4. Outcomes

The primary outcome used in this study was the change in patient's pain intensity as measured by the NPRS [23] and the change in the WOMAC [24] and AUSCAN [25] score for knee OA and hand OA, respectively, from baseline to 6 months.

The NPRS [23] is a one-dimensional scale for rating pain and has been recommended as a core outcome measure for chronic pain trials. Patients were asked to pick a discrete number from 0 to 10 (0 = no pain; 10 = worst pain imaginable) on the scale.

The WOMAC [24] OA index is a validated, self-administered, and responsive instrument widely used in clinical trials evaluating knee OA. It consists of 24 questions in three separated subscales: 5, pain, 2, stiffness, and 17, physical function. Each question is graded qualitatively, with a response rated from 0 to 4 points, and the WOMAC global scale is the sum of the three subscales (0 to 96). Higher scores indicate greater impact on quality of life.

The AUSCAN [25] comprises 15 items covering pain (*n* = 5), stiffness (*n* = 1), and function (*n* = 9). Response options for the AUSCAN items are on a five-point Likert scale (0–4) ranging from none to extreme. The total score is the sum of the responses of the 15 items (0–60). Higher scores on the AUSCAN indicate more pain and functional limitation.

Secondary outcomes included proportion of subjects who responded well to treatment, defined in accordance with the OMERACT-OARSI responder criteria [27] as ≥50% improvement in pain or function (high improvement), or ≥20% improvement in pain or function. Furthermore, the portion of patients taking rescue medicines at month 6 in the two groups was also analyzed.

### 2.5. Adverse Effects

Safety was assessed by analysis of adverse events (AEs). Patients were asked to report all AEs and cases with significant symptoms were assessed by detailed screening during the study. At the end of trial, a standardized interviewer-controlled questionnaire (Supplementary Table 1), including information on designated symptoms known to be probably associated with XLGB therapy (gastrointestinal disorders, hepatic disorders, renal function damage, and hypersensitivity) and information on other AEs by nonleading questions, was obtained. All AEs were recorded in detail based on the Common Terminology Criteria for Adverse Event v4.03 (Health and Services 2009).

We report adverse events that occurred during the main 6-month trial period, with onset on or after the first day of treatment and no later than 14 days after the last day of treatment.

### 2.6. Sample Size

Sample size for the two groups was calculated on the basis of conservative estimated that the average NPRS improvement level is 1.0 with a standard deviation of 2.0 according to the results of previous studies. The sample size was 85 for each group, which would provide a statistical power of more than 90% at a significance level of 0.05 (two-tailed). 10% dropout was estimated so that a total of 94 subjects were needed for each group. On the basis of our previous study showing the prevalence of symptomatic hand OA was about 7.8% in Chinese community people aged 40 or above, lower than knee OA; we screened about 2410 subjects for recruiting eligible patients.

### 2.7. Statistical Methods

Patients who received at least one dose of study medication were analyzed as Intention-To-Treat (ITT) population. Per-Protocol (PP) population was defined as the randomized patients who completed the study without major protocol violation. The demographic and safety data were analyzed using the ITT population. Efficacy analyses were performed using the ITT population and PP population. Missing data were processed using the Last Observation Carried Forward (LOCF) analysis.

Comparisons of baseline participant characteristics between XLGB and control groups used t-tests for continuous characteristics and Chi-square tests for categorical characteristics. An analysis of covariance (ANCOVA) model was used to test differences in primary outcome measures (mean changes) between treatment and control groups. The model included treatments, sex, rescue medicine usage status, K-L grades as fixed effects, with the body mass index (BMI), age, and baseline value of the relevant variable as covariates. The proportion of patients who responded well to treatments and the proportion of subjects taking rescue medicine between groups were compared using Chi-square test. Safety variables were summarized descriptively.

Analyses were performed using IBM SPSS Statistics version 20 (SPSS, IL, USA). For all tests *P* value < 0.05 was considered statistically significant.

## 3. Results

### 3.1. Participant Flow

**Figure 1:**
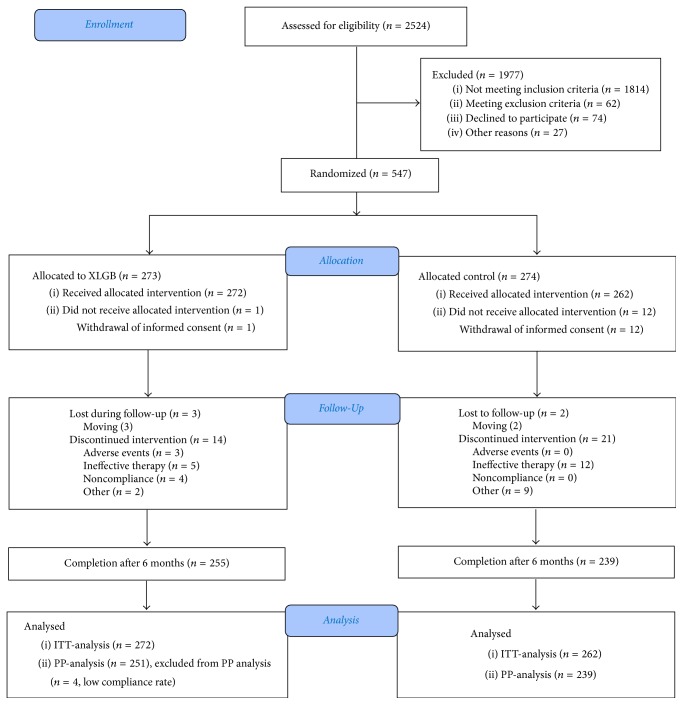
Flow diagram of clinical trial design.

Between August 2007 and October 2007, a total of 2524 potential participants from the 6 centers were screened. Among the 2524 persons, 1977 were excluded, including 1814 who did not meet the inclusion criteria, 62 who were within the exclusion criteria, 74 who declined to participate, and 27 with other reasons. 547 individuals were randomized (273 to XLGB and 274 to control group), and 13 did not receive allocated intervention. Of those 534 patients (272 to XLGB and 262 to control group) agreed to receive treatment, 255 (93.8%) in the XLGB group and 239 (91.2%) in the control arm completing the 6-month trial. The attrition rates were comparable between the intervention groups. Of the 494 patients who completed the clinical trial, 4 in XLGB group showed less than 70% adherence, which met the PP exclusion criteria resulting in a total of 490 patients for the PP analysis subject group (Figure 1).

### 3.2. Baseline Data

Patient demographics and baseline characteristics are summarized in Table 1. Mean age was 62.8 (SD, 10.0) years (range 40 to 87 years), and 321 of 534 patients (60.1%) were female. Mean BMI was 23.7 kg/m^2^ (SD, 3.17 kg/m^2^). No significant differences were observed between the two groups with regard to the age, sex, BMI, stage of OA (K-L grade), and the scale scores at baseline.

### 3.3. Primary Outcomes

For knee OA, the average improvement of NPRS in the XLGB and control groups compared to the baseline determined by the ITT analysis was 0.96 (SD, 2.14) and −0.93 (SD, 2.09), respectively, with significant improvement in XLGB group as compared with control group after 6 months' treatment (mean between-group difference, 1.89 [95% CI 1.52, 2.25]; *P* < 0.001); The average improvements in the WOMAC total scores in the XLGB and control groups compared to the baseline determined by the ITT analysis were 7.06 (SD, 13.67) and −3.00 (SD, 12.98), respectively, with significant statistical difference between groups (mean between-group difference, 10.06 [95% CI 7.86, 12.26]; *P* < 0.001). There also were greater reductions in WOMAC subscales in the XLGB group than in the control group (Table 2).

For hand OA, the average improvements of NPRS in the XLGB and control groups compared to the baseline were 0.50 (SD, 1.98) and −0.40 (SD, 1.84), respectively, with significant statistical difference between the two groups (mean between-group difference, 0.90 [95% CI 0.38, 1.90]; *P* = 0.001). The mean changes in AUSCAN total scores over 6 months were 4.60 (SD, 7.98) in the XLGB group and −2.16 (SD, 7.35) in the control group, which were significantly different (mean between-group difference, 6.76 [95% CI 4.75, 8.77]; *P* < 0.001), indicating a significant reduction in AUSCAN subscales in the XLGB group compared to the control group (Table 2).

Analysis stratified by sex and age (<60 versus ≥60) showed no differences between sex or age groups (Supplementary Tables  2 and 3).

### 3.4. Secondary Outcomes

The proportions of patients who responded to the treatment were higher in the XLGB group compared to the control group in patients with knee and hand OA (Table 2).

Patients took a range of rescue medications, but the most frequently used was analgesic (oral or topical). The proportions of patients taking rescue medicine were higher in the control group compared with XLGB group, in knee and hand OA patients (Table 2).

The PP analysis did not show differences in outcome data from the ITT analysis (Supplementary Table  4).

### 3.5. Potential Adverse Effects

AEs are summarized in Table 3. Overall, there were 25 AEs in 23 patients (8.5%) in the XLGB group based on ITT analysis. The most common adverse reaction in XLGB group was constipation followed by nausea, stomach discomfort, and dry mouth. All other AEs were each reported by only one patient (decreased appetite, abdominal pain, blood pressure increasing, vomiting, headache, and palpitation). All these AEs were considered moderate and possibly related to treatment. There were two serious AEs (SAEs) that led to withdrawal during the study in the XLGB group due to unrelated age-related diseases affecting completion of their follow-up assessment.

## 4. Discussion

This prospective, multicenter, randomized, controlled trial showed for the first time the efficacy of XLGB for controlling OA with respect to primary and secondary end points assessed at knee and hand. During the 6-month follow-up period, the pain and functional scores significantly improved in XLGB group compared to the control group. In addition, XLGB appeared to be safe and well accepted by patients over 6 months. Constipation, nausea, stomach discomfort, and dry mouth were the most commonly reported AEs. There were no SAEs that were considered to be treatment-related in the XLGB group.

This clinical trial showed both efficacy and safety of XLGB herbal formula for treatment of OA. In our study, the average improvement of pain and functional score in XLGB treatment group were significantly more compared to control group in knee and hand OA patients, and the proportion of patients who responded well to treatment were significantly higher in the XLGB group. However, the proportions of patients who responded well to treatment were lower in our study as compared with other studies of OA [28, 29]. One very important reason for this differences was that most of the previous studies recruited the participants from hospitals or clinics and a criterion for entry was a disease flare, while in our study participants were recruited from independent living communities and most of them were in the chronic course of OA with relatively mild symptoms and mild degree of pain. Furthermore, individualized treatment is fundamental to traditional Chinese medicines. Nevertheless, in the current study OA was diagnosed according to Western medicine instead of the theory of Chinese medicine, without subgroup analysis which was conducted according to Chinese medicine.

XLGB herbal formula has been used for centuries in China for treatment of bone disorders, including osteoporosis. In terms of TCM, symptoms of OA are usually known as “Bi” syndrome (bone rheumatism) or “flaccidity” (atrophic debility of bones) [30]. XLGB formula is based on the combination theory of herbs in Chinese medicine, which was commonly used to tone the “liver and kidney system” and strong bones and muscles [31]. It is clinically applicable to the treatment of diseases caused by deficiency of “liver and kidney system” and blood stasis and is consistent with the cause of “Bi” syndrome. A multicenter study demonstrated that XLGB treatment significantly increased BMD at lumbar spine and showed decline of bone turnover marker levels at month 6 in postmenopausal women [14], suggesting that OA patients especially females complicated with osteoporosis may benefit more from XLGB treatment. It also reflected the complexity of the phytochemistry of XLGB and its multiorgan responses. In a research by Dai et al., 118 compounds were identified or tentatively characterized from the original plants of XLGB using mass spectrometry method [26]. Another study on rat identified a total of 147 XLGB-related xenobiotics in rat biofluids after oral administration of XLGB [32]. The study also indicated that prenylated flavonol glycosides from Herba Epimedii, prenylated flavonoids from Fructus Psoraleae, saponins from Radix Dipsaci and Rhizoma Anemarrhenae, and tanshinones from Radix Salviae Miltiorrhizae were major absorbed chemical components of XLGB [32]. A recent study demonstrated that flavonoid compound icariin, the major bioactive component in Herba Epimedii, may serve as a hypoxia inducible factor- (HIF-) 1-alpha activator to promote articular cartilage repair through regulating chondrocyte proliferation, differentiation, and integration with subchondral bone formation [33]. In addition, as reported, icariin suppressed cartilage and bone degradation in mice of collagen-induced arthritis [34]. A study by Jung et al. found that administration of Radix Dipsaci extract in collagen-induced arthritis mice significantly reduced arthritic scores and serum levels of type II collagen antibody, prostaglandin E2 (PGE2), tumor necrosis factor- (TNF-) alpha, interleukin- (IL-) 1-beta, and IL-6, suggesting that Radix Dipsaci has anti-inflammatory and antiarthritic effects in arthritic mice [35]. In some respects, these results offer support for our findings. According to the latest medical knowledge, the inflammatory processes and immune system participated in the development and progression of OA as key elements in the pathogenesis of the disease [36, 37]. Previous studies in animal models have demonstrated anti-inflammatory and immunomodulatory effects of herbs* Salvia miltiorrhiza* and* Rehmannia glutinosa* Libosch [38, 39], which are two of seven herbal components of XLGB.* Salvia miltiorrhiza* has been shown to suppress the expression of inducible nitric oxide synthase (iNOS) and inhibited the production of oxygen free radicals, NO, IL-1-beta, and TNF-alpha [39, 40]. Similarly,* Rehmannia glutinosa* Libosch exhibited anti-inflammatory and antioxidative activities by inhibiting iNOS, Cyclooxygenase- (COX-) 2, IL-6, TNF-alpha, and monocyte chemoattractant protein-1 (MCP-1) [38, 41, 42]. The anti-inflammatory, antioxidative and immunomodulatory activities of these herbs may play an important role in XLGB exerting its therapeutic effect.

In current study, XLGB was safe and well tolerated by patients over 6 months, which was consistent with findings in previous reports. A study focusing on XLGB treatment effect on osteoporosis demonstrated that treatment with XLGB over one year was safe with no significant increase in the overall incidence of side effects compared to placebo [14]. Animal experimental study in rats also did not show toxic effects in the XLGB treated rats at doses up to 1000 mg/kg for 26 weeks, except for a slight increase in the organ weight of the uterus at doses higher than 300 mg/kg [16]. In fact, XLGB has had a long prescription history for postmenopausal women in China, and no persistent adverse events related to XLGB administration was reported in the past 15 years according to the CFDA database (http://eng.sfda.gov.cn/WS03/CL0755/).

There were some limitations to this study. First, the study was an open-label design and the control group patients did not take a placebo. Patients in this study were not blinded to treatment allocation and neither were clinical staff or researchers who performed outcomes assessment. Another limitation was a rather short duration of follow-up where 6-month treatment period was not long enough to see a beneficial effect on long term. In addition, the single visit after treatment at month 6 did not provide enough information of early period of treatment, such as weeks 6 and 12. Last, there was no biochemical monitoring for adverse events, although it demonstrated that multiple components of XLGB underwent comprehensive hepatobiliary excretion [30] and previous study reported that XLGB treatment might be related to temporal liver enzyme abnormality that was however not statistically significant as compared with placebo control group [14].

## 5. Conclusion

In conclusion, the present study showed that XLGB was effective in reducing pain and related symptoms and to improve function of the knee and hand in patients with OA over a 6-month period, suggesting its potential as an alternative treatment for OA. Future studies should explore the long term benefits.

## Figures and Tables

**Table 1:** Participant demographics and baseline characteristics.

Characteristics	Total	XLGB	Control	*P value*
*Number of subjects*	534	272	262	
*Age*				
Mean (SD), y	62.51 (10.05)	62.40 (9.87)	62.63 (10.26)	0.793
≥60, number (%)	314 (58.8)	162 (59.6)	152 (58.0)	0.717
*Gender *				
Female, number (%)	321 (60.1)	162 (59.6)	159 (60.7)	0.790
*Residence *				
Urban, number (%)	259 (48.5)	136 (50.0)	123 (46.9)	0.480
Rural, number (%)	275 (51.5)	136 (50.0)	139 (53.1)	
*Education*				
Primary school or less	274 (51.3)	136 (50.0)	138 (52.7)	0.730
Middle school	151 (28.3)	77 (28.3)	74 (28.2)	
High school or more	109 (20.4)	59 (21.7)	50 (19.1)	
*Current smoking*, number (%)	122 (22.8)	64 (23.5)	55 (21.0)	0.481
*Current alcohol consumption*, number (%)	101 (18.9)	52 (19.1)	51 (19.5)	0.919
*BMI*				
<25, number (%)	355 (66.5)	177 (65.1)	178 (67.9)	0.746
25~30, number (%)	157 (29.4)	84 (30.9)	73 (27.9)	
≥30, number (%)	22 (4.1)	11 (4.0)	11 (4.2)	
*Knee OA*				
Number	454	230	224	
K-L grades				
K-L 2, number (%)	212 (46.7)	111 (48.3)	101 (45.1)	0.664
K-L 3, number (%)	187 (41.2)	90 (39.1)	97 (43.3)	
K-L 4, number (%)	55 (12.1)	29 (12.6)	26 (11.6)	
NPRS, mean (SD)	4.85 (1.87)	4.83 (1.83)	4.87 (1.91)	0.819
WOMAC score				
Total, mean (SD)	43.51 (13.81)	43.40 (13.31)	43.61 (14.33)	0.873
Pain subscale, mean (SD)	11.31 (3.64)	11.28 (3.50)	11.33 (3.78)	0.879
Stiffness subscale, mean (SD)	4.06 (1.58)	4.03 (1.48)	4.09 (1.67)	0.670
Function subscale, mean (SD)	28.14 (8.74)	28.10 (8.49)	28.19 (9.01)	0.911
*Hand OA*				
Number	181	91	90	
K-L grades				
K-L 2, number (%)	121 (66.9)	58 (63.7)	63 (70.0)	0.663
K-L 3, number (%)	45 (24.9)	25 (27.5)	20 (22.2)	
K-L 4, number (%)	15 (8.3)	8 (8.8)	7 (7.8)	
NPRS, mean (SD)	4.55 (13.81)	4.57 (1.83)	4.52 (1.74)	0.853
AUSCAN score				
Total, mean (SD)	27.51 (9.33)	27.70 (9.13)	27.32 (9.58)	0.784
Pain subscale, mean (SD)	9.23 (3.13)	9.25 (3.13)	9.20 (3.14)	0.910
Stiffness subscale, mean (SD)	1.96 (0.78)	1.95 (0.77)	1.98 (0.79)	0.788
Function subscale, mean (SD)	16.33 (5.81)	16.51 (5.75)	16.14 (5.90)	0.677

XLGB: Xianlinggubao herbal formula; BMI: body mass index; OA: osteoarthritis; K-L: Kellgren and Lawrence grade; NPRS: numeric pain rating scale; WOMAC: Western Ontario and McMaster Universities Arthritis Index; AUSCAN: Australian/Canadian Osteoarthritis Hand Index.

**Table 2:** Primary and secondary outcomes after 6 months' intervention.

	XLGB	Control	Estimated treatment difference, XLGB versus control (95% CI)^†^	*P*
*Knee OA*				
NPRS (0–10)				
Change from baseline, mean (SD)	−0.96 (2.14)	0.93 (2.09)	1.89 (1.52, 2.25)	**<0.001**
50% decrease, number (%)	83 (36.1)	19 (8.5)		**<0.001**
20% decrease, number (%)	165 (71.7)	45 (20.1)		**<0.001**
WOMAC (0–96)				
Total				
Change from baseline, mean (SD)	−7.06 (13.67)	3.00 (12.98)	10.06 (7.86, 12.26)	**<0.001**
Pain subscale				
Change from baseline, mean (SD)	−2.25 (4.03)	0.42 (3.83)	2.67 (2.02, 3.32)	**<0.001**
50% decrease, number (%)	55 (23.9)	12 (5.4)		**<0.001**
20% decrease, number (%)	136 (59.1)	42 (18.8)		**<0.001**
Stiffness subscale				
Change from baseline, mean (SD)	−0.46 (1.44)	0.48 (1.42)	0.93 (0.69, 1.18)	**<0.001**
Function subscale				
Change from baseline, mean (SD)	−4.35 (8.25)	2.09 (7.78)	6.44 (5.12, 7.76)	**<0.001**
50% decrease, number (%)	42 (18.3)	10 (4.5)		**<0.001**
20% decrease, number (%)	122 (53.0)	38 (17.0)		**<0.001**
Patients taking rescue medicine				
Number (%)	13 (5.7)	50 (22.3)		**<0.001**
*Hand OA*				
NPRS (0–10)				
Change from baseline, mean (SD)	−0.50 (1.98)	0.40 (1.84)	0.90 (0.38, 1.90)	**=0.001**
50% decrease, number (%)	25 (27.5)	11 (12.2)		**=0.010**
20% decrease, number (%)	45 (49.5)	20 (22.2)		**<0.001**
AUSCAN (0–60)				
Total				
Change from baseline, mean (SD)	−4.60 (7.98)	2.16 (7.35)	6.76 (4.75, 8.77)	**<0.001**
Pain subscale				
Change from baseline, mean (SD)	−1.61 (3.44)	1.01 (3.17)	2.61 (1.73, 3.50)	**<0.001**
50% decrease, number (%)	22 (24.2)	4 (4.4)		**<0.001**
20% decrease, number (%)	45 (49.5)	10 (11.1)		**<0.001**
Stiffness subscale				
Change from baseline, mean (SD)	−0.18 (0.52)	0.12 (0.48)	0.30 (0.16, 0.44)	**<0.001**
Function subscale				
Change from baseline, mean (SD)	−2.78 (4.10)	1.04 (3.83)	3.82 (2.76, 4.87)	**<0.001**
50% decrease, number (%)	11 (12.1)	3 (3.3)		**=0.027**
20% decrease, number (%)	44 (48.4)	10 (11.1)		**<0.001**
Patients taking rescue medicine				
Number (%)	7 (7.7)	21 (23.3)		**=0.004**

^†^Estimated differences in treatment effect are from an analysis of covariance with data from the ITT population, with last-observation-carried-forward (LOCF) imputation. The ITT population comprising patients who underwent randomization were exposed to at least one treatment dose. XLGB: Xianlinggubao herbal formula; OA: osteoarthritis; NPRS: numeric pain rating scale; WOMAC: Western Ontario and McMaster Universities Arthritis Index; AUSCAN: Australian/Canadian Osteoarthritis Hand Index.

**Table 3:** Adverse events documented during the 6-month trial period in the ITT population.

Events	XLGB (*n* = 272)
*Adverse events*	
Total participants with any adverse events (%)	23^*∗*^
Constipation	9
Nausea	3
Stomach discomfort	2
Dry mouth	2
Decreased appetite	1
Abdominal pain	1
Blood pressure increasing	1
Vomiting	1
Headache	1
Palpitation	1
*Serious adverse events*	
Death (myocardial infarction)	1
Pneumonia	1

^*∗*^One participant had constipation and stomach discomfort and another participant had constipation and dry mouth.

## Supplementary Materials

Supplementary Materials Supplementary Table  1: documentation table of adverse effects of drug administration. Supplementary Table  2: primary outcomes after 6 months' intervention, stratified by sex. Supplementary Table  3: primary outcomes after 6 months' intervention, stratified by age. Supplementary Table  4: primary and secondary outcomes after 6 months' intervention, the PP analysis.
