# Supplementary material for: A Traditional Herbal Formula Xianlinggubao for Pain Control and Function Improvement in Patients with Knee and Hand Osteoarthritis: A Multicenter, Randomized, Open-Label, Controlled Trial
Source: Evid Based Complement Alternat Med. 2018 Feb 12;2018:1827528. doi: 10.1155/2018/1827528 (PMC5829359; doi:10.1155/2018/1827528)
Supplement: Supplementary Materials — Supplementary Table 1: documentation table of adverse effects of drug administration. Supplementary Table 2: primary outcomes after 6 months' intervention, stratified by sex. Supplementary Table 3: primary outcomes after 6 months' intervention, stratified by age. Supplementary Table 4: primary and secondary outcomes after 6 months' intervention, the PP analysis. [file 1827528.f1.docx]

Supplementary Table 1. Documentation Table of Adverse Effects of Drug Administration

| **Adverse Drug Reaction** | | **Symptom description** | **Time of occurrence** | **Management** | | | **Consequence** | **Time of adverse reactions disappear** | **Notes** |
| --- | --- | --- | --- | --- | --- | --- | --- | --- | --- |
|  |  |  |  | **No treatment** | **Suspend medication ( and Observation time)** | **Treatment with drug (what kind of drug and Drug use time)** |  |  |  |
| Gastrointestinal reactions | |  |  |  |  |  |  |  |  |
| Allergic reaction (rash) | |  |  |  |  |  |  |  |  |
| Liver and kidney function abnormalities | |  |  |  |  |  |  |  |  |
| others |  |  |  |  |  |  |  |  |  |
|  |  |  |  |  |  |  |  |  |  |
|  |  |  |  |  |  |  |  |  |  |
| Notes | |  | | | | | | | |

Supplementary Table 2. Primary outcomes after 6 months intervention, stratified by sex.

|  | XLGB | Control | Estimated difference in treatment effect, XLGB vs. Control (95% CI)† | *P* |
| --- | --- | --- | --- | --- |
| Men |  |  |  |  |
| **Knee OA** |  |  |  |  |
| Number | 94 | 85 |  |  |
| Change from baseline, mean (SD) |  |  |  |  |
| NPRS (0-10) | -0.95 (1.94) | 0.70 (2.16) | 1.65 (1.08, 2.23) | **<0.001** |
| WOMAC (0-96) |  |  |  |  |
| Total | -7.14 (12.15) | 1.61 (14.21) | 8.75 (5.34, 12.16) | **<0.001** |
| Pain subscale | -2.20 (3.58) | 0.08 (4.18) | 2.28 (1.28, 3.29) | **<0.001** |
| Stiffness subscale | -0.41 (1.28) | 0.37 (1.56) | 0.78 (0.40, 1.17) | **<0.001** |
| Function subscale | -4.53 (7.33) | 1.15 (8.52) | 5.68 (3.63, 7.73) | **<0.001** |
| **Hand OA** |  |  |  |  |
| Number | 32 | 33 |  |  |
| Change from baseline, mean (SD) |  |  |  |  |
| NPRS (0-10) | -0.55 (2.04) | 0.40 (1.73) | 0.95 (0.13, 1.88) | **=0.047** |
| AUSCAN (0-60) |  |  |  |  |
| Total | -5.04 (8.39) | 1.65 (8.02) | 6.69 (2.80, 10.57) | **=0.001** |
| Pain subscale | -1.71 (3.61) | 0.78 (3.53) | 2.48 (0.78, 4.19) | **=0.005** |
| Stiffness subscale | -0.24 (0.55) | 0.02 (0.56) | 0.25 (-0.09, 0.55) | =0.089 |
| Function subscale | -3.07 (4.33) | 0.86 (4.05) | 3.93 (1.87, 5.98) | **<0.001** |
|  |  |  |  |  |
| Women |  |  |  |  |
| **Knee OA** |  |  |  |  |
| Number | 136 | 139 |  |  |
| Change from baseline, mean (SD) |  |  |  |  |
| NPRS (0-10) | -0.90 (2.27) | 1.11 (2.04) | 2.01 (1.53, 2.48) | **<0.001** |
| WOMAC (0-96) |  |  |  |  |
| Total | -6.49 (14.64) | 4.23 (12.20) | 10.77 (7.85, 13.68) | **<0.001** |
| Pain subscale | -2.12 (4.32) | 0.75 (3.61) | 2.87 (2.00, 3.74) | **<0.001** |
| Stiffness subscale | -0.44 (1.53) | 0.58 (1.34) | 1.01 (0.69, 1.34) | **<0.001** |
| Function subscale | -3.92 (8.83) | 2.94 (7.31) | 6.86 (5.12, 8.61) | **<0.001** |
| **Hand OA** |  |  |  |  |
| Number | 59 | 57 |  |  |
| Change from baseline, mean (SD) |  |  |  |  |
| NPRS (0-10) | -0.35 (1.91) | 0.53 (1.88) | 0.88 (0.23, 1.53) | **=0.008** |
| AUSCAN (0-60) |  |  |  |  |
| Total | -3.50 (7.64) | 3.30 (6.87) | 6.80 (4.37, 9.23) | **<0.001** |
| Pain subscale | -1.15 (3.31) | 1.49 (2.93) | 2.64 (1.56, 3.71) | **<0.001** |
| Stiffness subscale | -0.08 (0.50) | 0.22 (0.41) | 0.30 (0.14, 0.47) | **<0.001** |
| Function subscale | -2.24 (3.93) | 1.59 (3.67) | 3.83 (2.56, 5.10) | **<0.001** |

† Estimated difference in treatment effect are from an analysis of covariance with data from the ITT population, with last-observation-carried-forward (LOCF) imputation. The ITT population comprised patients who underwent randomization, were exposed to at least one treatment dose.

XLGB: Xianlinggubao herbal formula; OA: osteoarthritis; NPRS: numeric pain rating scale; WOMAC: Western Ontario and McMaster Universities Arthritis Index; AUSCAN: Australian/Canadian Osteoarthritis Hand Index.

Supplementary Table 3. Primary outcomes after 6 months intervention, stratified by age.

|  | XLGB | Control | Estimated difference in treatment effect, XLGB vs. Control (95% CI)† | *P* |
| --- | --- | --- | --- | --- |
| <60 years old |  |  |  |  |
| **Knee OA** |  |  |  |  |
| Number | 92 | 87 |  |  |
| Change from baseline, mean (SD) |  |  |  |  |
| NPRS (0-10) | -0.58 (1.77) | 1.61 (2.16) | 2.19 (1.65, 2.74) | **<0.001** |
| WOMAC (0-96) |  |  |  |  |
| Total | -3.56 (11.68) | 7.74 (13.59) | 11.30 (7.86, 14.75) | **<0.001** |
| Pain subscale | -1.21 (3.44) | 1.82 (4.01) | 3.03 (2.02, 4.05) | **<0.001** |
| Stiffness subscale | -0.15 (1.23) | 0.90 (1.50) | 1.05 (0.66, 1.43) | **<0.001** |
| Function subscale | -2.20 (7.06) | 5.00 (8.13) | 7.20 (5.14, 9.26) | **<0.001** |
| **Hand OA** |  |  |  |  |
| Number | 29 | 40 |  |  |
| Change from baseline, mean (SD) |  |  |  |  |
| NPRS (0-10) | -0.22 (2.11) | 0.65 (1.53) | 0.87 (0.10, 1.64) | **=0.028** |
| AUSCAN (0-60) |  |  |  |  |
| Total | -3.94 (7.84) | 1.36 (6.37) | 5.30 (2.46, 8.14) | **<0.001** |
| Pain subscale | -1.34 (3.46) | 0.70 (2.70) | 2.04 (0.77, 3.30) | **=0.002** |
| Stiffness subscale | -0.20 (0.52) | 0.01 (0.35) | 0.22 (0.02, 0.41) | **=0.032** |
| Function subscale | -2.38 (3.95) | 0.69 (3.49) | 3.07 (1.55, 4.59) | **<0.001** |
|  |  |  |  |  |
| ≥60 years old |  |  |  |  |
| **Knee OA** |  |  |  |  |
| Number | 138 | 137 |  |  |
| Change from baseline, mean (SD) |  |  |  |  |
| NPRS (0-10) | -1.12 (2.36) | 0.54 (2.04) | 1.67 (1.16, 2.17) | **<0.001** |
| WOMAC (0-96) |  |  |  |  |
| Total | -8.40 (14.88) | 0.84 (12.61) | 9.24 (6.26, 12.22) | **<0.001** |
| Pain subscale | -2.65 (4.40) | -0.21 (3.72) | 2.45 (1.56, 3.33) | **<0.001** |
| Stiffness subscale | -0.58 (1.56) | 0.27 (1.38) | 0.85 (0.52, 1.19) | **<0.001** |
| Function subscale | -5.16 (8.96) | 0.78 (7.57) | 5.94 (4.15, 7.73) | **<0.001** |
| **Hand OA** |  |  |  |  |
| Number | 62 | 50 |  |  |
| Change from baseline, mean (SD) |  |  |  |  |
| NPRS (0-10) | -0.68 (1.91) | 0.21 (2.05) | 0.89 (0.17, 1.61) | **=0.016** |
| AUSCAN (0-60) |  |  |  |  |
| Total | -5.54 (7.93) | 2.34 (8.11) | 7.88 (5.05, 10.70) | **<0.001** |
| Pain subscale | -1.98 (3.40) | 1.10 (3.54) | 3.08 (1.83, 4.33) | **<0.001** |
| Stiffness subscale | -0.21 (0.52) | 0.14 (0.56) | 0.35 (0.15, 0.55) | **=0.001** |
| Function subscale | -3.32 (4.10) | 1.08 (4.11) | 4.40 (2.92, 5.87) | **<0.001** |

† Estimated differences in treatment effects are from an analysis of covariance with data from the ITT population, with last-observation-carried-forward (LOCF) imputation. The ITT population comprised patients who underwent randomization, were exposed to at least one treatment dose.

XLGB: Xianlinggubao herbal formula; OA: osteoarthritis; NPRS: numeric pain rating scale; WOMAC: Western Ontario and McMaster Universities Arthritis Index; AUSCAN: Australian/Canadian Osteoarthritis Hand Index.

Supplementary Table 4. Primary and secondary outcomes after 6 months intervention, the PP analysis.

|  | XLGB | Control | Estimated difference in treatment effect, XLGB vs. Control (95% CI)† | *P* |
| --- | --- | --- | --- | --- |
| **Knee OA** |  |  |  |  |
| Number | 213 | 201 |  |  |
| NPRS (0-10) |  |  |  |  |
| Change from baseline, mean (SD) | -0.96 (2.21) | 0.93 (2.16) | 1.88 (1.49, 2.28) | **<0.001** |
| 50% decrease, No. (%) | 81 (38.0) | 18 (9.0) |  | **<0.001** |
| 20% decrease, No. (%) | 157 (73.7) | 42 (20.9) |  | **<0.001** |
| WOMAC (0-96) |  |  |  |  |
| Total |  |  |  |  |
| Change from baseline, mean (SD) | -7.39 (13.95) | 3.24 (13.70) | 10.63 (8.26, 13.00) | **<0.001** |
| Pain subscale |  |  |  |  |
| Change from baseline, mean (SD) | -2.34 (4.11) | 0.48 (4.04) | 2.82 (2.12, 3.52) | **<0.001** |
| 50% decrease, No. (%) | 55 (25.8) | 12 (6.0) |  | **<0.001** |
| 20% decrease, No. (%) | 135 (63.4) | 42 (20.9) |  | **<0.001** |
| Stiffness subscale |  |  |  |  |
| Change from baseline, mean (SD) | -0.49 (1.47) | 0.52 (1.50) | 1.01 (0.74, 1.27) | **<0.001** |
| Function subscale |  |  |  |  |
| Change from baseline, mean (SD) | -4.56 (8.40) | 2.23 (8.21) | 6.79 (5.37, 8.21) | **<0.001** |
| 50% decrease, No. (%) | 42 (19.7) | 10 (5.0) |  | **<0.001** |
| 20% decrease, No. (%) | 122 (57.3) | 38 (18.9) |  | **<0.001** |
| Patients taking rescue medicine |  |  |  |  |
| No. (%) | 11 (5.2) | 47 (23.4) |  | **<0.001** |
| **Hand OA** |  |  |  |  |
| Number | 88 | 86 |  |  |
| NPRS (0-10) |  |  |  |  |
| Change from baseline, mean (SD) | -0.50 (1.97) | 0.39 (1.87) | 0.89 (0.36, 1.42) | **=0.001** |
| 50% decrease, No. (%) | 25 (28.4) | 11 (12.8) |  | **=0.011** |
| 20% decrease, No. (%) | 45 (51.1) | 19 (22.1) |  | **<0.001** |
| AUSCAN (0-60) |  |  |  |  |
| Total |  |  |  |  |
| Change from baseline, mean (SD) | -4.74 (8.00) | 2.13 (7.37) | 6.87 (4.80, 8.94) | **<0.001** |
| Pain subscale |  |  |  |  |
| Change from baseline, mean (SD) | -1.65 (3.46) | 0.99 (3.20) | 2.64 (1.72, 3.56) | **<0.001** |
| 50% decrease, No. (%) | 22 (25.0) | 4 (4.7) |  | **<0.001** |
| 20% decrease, No. (%) | 45 (51.1) | 10 (11.6) |  | **<0.001** |
| Stiffness subscale |  |  |  |  |
| Change from baseline, mean (SD) | -0.19 (0.53) | 0.10 (0.48) | 0.29 (0.14, 0.44) | **<0.001** |
| Function subscale |  |  |  |  |
| Change from baseline, mean (SD) | -2.87 (4.11) | 1.04 (3.82) | 3.90 (2.82, 4.99) | **<0.001** |
| 50% decrease, No. (%) | 11 (12.5) | 3 (3.5) |  | **=0.029** |
| 20% decrease, No. (%) | 44 (50.0) | 9 (10.5) |  | **<0.001** |
| Patients taking rescue medicine |  |  |  |  |
| No. (%) | 7 (8.0) | 20 (23.3) |  | **=0.005** |

† Estimated differences in treatment effects are from an analysis of covariance with data from the PP population, the PP population was defined as the randomized patients who completed the study without major protocol violation.

XLGB: Xianlinggubao herbal formula; OA: osteoarthritis; NPRS: numeric pain rating scale; WOMAC: Western Ontario and McMaster Universities Arthritis Index; AUSCAN: Australian/Canadian Osteoarthritis Hand Index.
